# Time and chemotherapy treatment trends in the treatment of elderly patients (age ⩾70 years) with small cell lung cancer

**DOI:** 10.1038/sj.bjc.6602888

**Published:** 2005-11-29

**Authors:** T Yau, S Ashley, S Popat, A Norton, A Matakidou, J Coward, M E R O'Brien

**Affiliations:** 1 Lung Unit, Royal Marsden Hospital, Downs Road, Sutton, Surrey SM2 5PT, UK

**Keywords:** age and cancer, chemotherapy, lung cancer

## Abstract

Platinum-based treatment for small cell lung cancer (SCLC) has been established since 1995. This study investigates treatment outcome of elderly patients (age ⩾70 years) with SCLC over the past 20 years in a large UK cancer centre. Comparison of all-cause survival was assessed in patients presenting between two predefined time periods: 1982–1994 and 1995–2003. All the survival analysis were adjusted for stage and performance status and age if appropriate. Survival between different chemotherapy treatment regimens was compared. A total of 322 elderly patients (31% of all) registered between 1982–2003 received chemotherapy for SCLC. Patients presenting in 1995–2003 had an overall better median survival (43 *vs* 25 weeks) and a 1-year survival (37 *vs* 14%) than patients presenting in 1982–1994 (*P*<0.001). This applied to patients with both limited and extensive stage disease and all age groups. There was a trend towards the use of more platinum-based treatments in the later cohort but the use of radiotherapy remained constant. Patients who received platinum combinations (Carboplatin or Cisplatin) had significantly improved survival over those who received single agents or other combinations (*P*<0.001) and there was no significant difference between carboplatin and cisplatin (*P*=0.7). The analysis demonstrates that there has been a significant improvement in survival for elderly patients with lung cancer treated by chemotherapy in the past 20 years despite more very elderly patients being treated with a poorer performance status. This change is probably multifactorial and may be due to the increased use of platinum-based treatment and improved supportive care.

Lung cancer is the main cause of cancer deaths worldwide. The median age at diagnosis in developed countries is approximately 68 years, and as many as 40% of patients may be older than 70 years (Bunn and Lilenbaum, 2003). In the UK, lung cancer is primarily a disease of the elderly, with a peak incidence between the ages of 70 and 80 years (Cancer Research Campaign, 2001). In the treatment of small cell lung cancer (SCLC), there has been a move to platinum-based treatment (Sundstrom *et al*, 2002). Local thoracic radiotherapy and prophylactic cranial irradiation now have a place not only in decreasing local recurrence but also in prolonging survival (Pignon *et al*, 1992; Auperin *et al*, 1999). Compared to the younger age group, elderly patients have significantly more comorbidity, particularly from pulmonary and cardiovascular disease, and may also demonstrate differences in drug pharmacokinetics. Chemotherapy is often withheld from elderly patients due to the fear of potential toxicities and modest survival advantage (Earle *et al*, 2000; Bunn, 2002). In addition, there are little published efficacy data for chemotherapy in this patient group, as they have generally been under-represented in clinical trials (Hutchins *et al*, 1999). This study evaluates the survival time trends and chemotherapy outcomes in elderly patients with SCLC, greater than or equal to 70 years of age, treated at a single institution over a 20-year period.

## PATIENTS AND METHODS

### Patients

Data on all SCLC patients receiving chemotherapy at the Royal Marsden Hospital (RMH) from 1982 to 2003 were prospectively entered and updated on an electronic database. Recorded data included demographic information, date of presentation to the RMH, stage of disease, performance status (PS), treatment details, and survival status. The diagnosis of SCLC was based on histological or cytological features of the tumour. Tumour tissue was reviewed by a central pathologist until 1998 when central review was discontinued after an internal audit showed no change in diagnosis with review. Thereafter, pathology diagnosis from the original referring hospital was accepted unless clinical picture was inconsistent with referring diagnosis.

Staging was based on both clinical and radiological (CXR and CT scanning) features and was classified according to limited and extensive stage disease defined by the Veterans Administration Lung Study Group (Zelen, 1973). Performance status of patients was graded according to World Health Organization criteria (World Health Organisation, 1979).

Chemotherapy was offered to most patients with SCLC over the 20-year period. Carboplatin was used instead of cisplatin if patients had poor renal function, deafness, could not tolerate a fluid challenge or could not stay in hospital for the duration of cisplatin pre- and posthydration. Patients were excluded from analysis if they had not had adequate follow-up information or no histological diagnosis.

### Statistical analysis

Survival was analysed by means of the proportional hazards model (Cox, 1972) and was adjusted for the prognostic factors of stage and PS.

Two predefined time periods, that is patients presenting between 1982–1994 and between 1995–2003 was used for comparison of survival. The year 1995 was chosen as a cutoff point because platinum-based treatment was introduced around this time to treat both limited and extensive stage SCLC patients. Survival by time period, and also by treatment regimen, was adjusted for stage, PS and age. In view of the changes in the local treatment guidelines for elderly patients between the two time periods and the three age groups, imbalances were tested for by means of the Fisher's exact test and the Mann–Whitney (MW) test. Survival was analysed by means of the Cox's proportional hazards model and was adjusted for the prognostic factors of stage and PS.

## RESULTS

### Demographics

Between 1982 and December 2003, 1163 SCLC patients were recorded on the lung unit database. In all, 802 (69%) of these patients were aged <70 years and 361 (31%) patients aged 70 years or older. Of the elderly patients, 39 did not receive chemotherapy and they have been excluded from the analysis. This report focuses on the remaining 322 elderly patients (age ⩾70 years), of which 157 patients presented between 1982 and 1994, and 165 patients between 1995 and 2003.

Table 1 demonstrates patient demographic data stratified by year of presentation. In all, 188 patients (58%) were aged 70–74 years, 108 (34%) were aged 75–79 years and 26 (8%) were aged ⩾80 years. The proportion of all patients over the age of 80 years increased from 6 to 10% between the two time periods. The PS of elderly patients treated in 1995–2003 was overall poorer than those presenting before 1995.

Demographic and treatment data for patients with SCLC is shown for the three age groups in Table 2. The proportion of extensive disease patients treated with chemotherapy decreased with the age group (*P*=0.007; MW test). Patients in the 70- to 74-year-age group had a better PS than older patients (*P*=0.005; MW test). Table 3 shows the treatment modalities stratified by time period. There was increased use of platinum compounds over time (1982–94: 48%, 1995–2003: 75%). Similar numbers of patients received thoracic and/or prophylactic cranial irradiation.

### Survival

**Figure 1:**
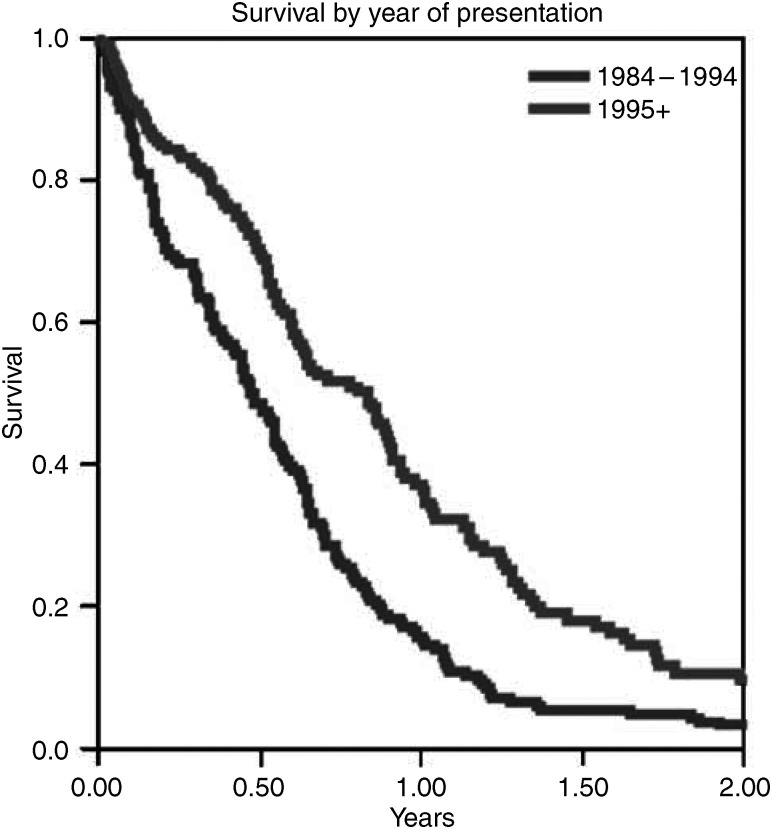
Survival by year of presentation graph.

Overall, there has been an improvement in survival after adjusting for stage, PS and age. Patients presenting in 1995–2003 had an overall better median survival (43 *vs* 25 weeks) and a 1-year survival (37 *vs* 14%) than patients presenting in 1982–1994 (*P*<0.001, Figure 1). This applied to patients with both limited and extensive stage disease. For limited stage 1995–2003 the median survival was 59 weeks compared to 27 in 1982–1994 (*P*<0.001) and for extensive disease the same trend was seen with median survivals of 39 *vs* 23 weeks (*P*=0.02). Table 4 demonstrates the improvement in survival in different elderly age groups. This survival difference was significant in the 70- to 74-year-age group (*P*=0.001) but, possibly due to small numbers, the difference did not reach significance in the older age groups.

### Survival according to chemotherapy treatment

**Figure 2:**
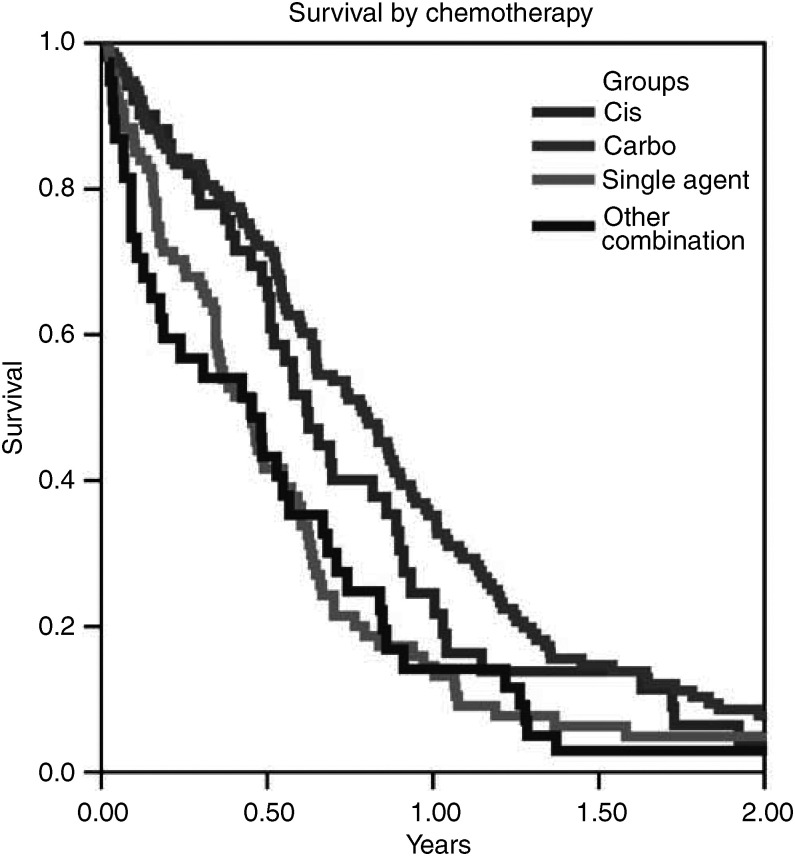
Survival by treatment.

Patients who received platinum combinations (carboplatin or cisplatin) had significantly improved survival over those who received single agents or other combinations (*P*<0.001) (Figure 2). There was no significant difference between carboplatin and cisplatin (*P*=0.7).

## DISCUSSION

This study suggests that there has been a significant improvement in survival for SCLC elderly patients (age ⩾70 years) receiving treatment over the past 20 years despite the trend to treat more of the very elderly and patients with a worse PS. This improvement is still present when patients are controlled for sex, stage and PS, suggesting that this is due to advances in patient management adjunctive therapies and in particular the use of platinum (and carboplatin)-based therapy rather than earlier presentation of the disease.

In SCLC, combination chemotherapy has been an established treatment for decades. In contrast to elderly patients with NSCLC, the majority of elderly patients with SCLC will receive active treatment (chemotherapy and radiotherapy) – only 39/361 of our patients did not receive treatment (Brown *et al*, 1996). Pooled data of the Southwest Oncology Group (Albain *et al*, 1990) and CALGB (Spiegelman *et al*, 1989) showed that age was a significant predictor favouring the younger patient for both limited and extensive disease SCLC although a large French retrospective review of SCLC patients using numerous regimens found no such correlation (Lebitasy *et al*, 2001). Our median survivals for all these elderly patients are still in the range of reported studies. Single-agent chemotherapy, namely oral etoposide, was initially thought to be a reasonable treatment for elderly patients with SCLC (Smit *et al*, 1989). However, two large randomised trials demonstrated inferior survival when this regime was compared to combination chemotherapy (Girling, 1996; Souhami *et al*, 1997) and our data would support this in an nontrial setting. In limited stage SCLC, Siu *et al* (1996) demonstrated that combination chemotherapy has similar efficacy in both young and elderly patients and Murray *et al* (1998) showed that treatment toxicities may be minimised while maintaining similar outcomes by giving two cycles of chemotherapy followed by radiotherapy, rather than a longer course of chemotherapy. However, subset analysis of an Intergroup trial found that elderly patients (age > 70 years) obtained similar responses and survival figures to younger patients and that the elderly subgroup did significantly worse when treated by only two cycles of chemotherapy (Yuen *et al*, 2000). There was no major change in the number of cycles given in our populations over time. In extensive stage SCLC, Pujol *et al* (2001) found that elderly patients (age > 70 years) had similar response rates and survival to young patients when they were treated by cisplatin combinations and there was no statistically significant impact of age on these outcomes.

Platinum-based treatment with etoposide has been our treatment of choice for all SCLC patients since around 1995. Outcome improvements in SCLC patients are not restricted to any particular stage or age group. Given that carboplatin was generally used when patients were not considered fit enough for cisplatin the results with carboplatin are very reassuring and the carboplatin etoposide is now the standard in the elderly in our unit. While the benefits of radiotherapy on local disease control have been known for 20 years, the survival benefits of radiotherapy are a more recent addition to the general consensus. (Auperin *et al*, 1999). In our study, the impact of radiotherapy on the differences in survival between the two cohorts in SCLC patients is negligible as there were a similar proportion of patients who received thoracic and/or prophylactic cranial irradiation between the two cohorts. There are few data in the literature reporting the outcomes of chemotherapy treatment in elderly patients with SCLC. Most studies available include elderly patients with SCLC up to 75 years old. In our study, we have analysed the outcome of 322 elderly patients older than 70 years, and 134 patients older than 75 years with SCLC. This is the largest series in the literature so far.

This improvement in treatment outcomes overall might also be attributed to other factors. Changes in guidelines and different working procedures have resulted in a very large percentage of patients with lung cancer being referred for oncological assessment. Better assessment of comorbidity and PS of patients has resulted in better selection of patients for chemotherapy. There is now better supportive care, such as antiemetics, laxatives, antibiotics, etc. available for these patients, so that courses of treatment of appropriate intensity and duration can be safely administered. The contribution of nutritional status, social and family support are of course not easily quantifiable and difficult to evaluate over a long time period but may also be relevant. Last but not least, advances in the medical oncology treatment of lung cancer, including the use of second-line therapies may also have played a role in the improvement in survival over the recent years in this elderly population (age ⩾70 years).

In conclusion, the outcome for elderly patients with SCLC in our sector has globally improved. In our experience, carboplatin combinations have as good an outcome and better toxicity profile than other combinations or nonplatinum single agents chemotherapy in the elderly with SCLC.

## Figures and Tables

**Table 1:** Demographic data stratified by year of presentation (SCLC treated)

	**No. of patients (%)**		
	**1982–1994**	**1995–2003**	**Total**
Patients	157 (49)	165 (51)	322

*Sex*			
Male	95 (64)	95 (58)	190 (59)
Female	62 (39)	70 (42)	132 (41)

*Age (years)*			
70–74	102 (65)	86 (52)	188 (58)
75–79	45 (29)	63 (38)	108 (34)
80+	10 (6)	16 (10)	26 (8)

*Stage*			
Limited	76 (48)	88 (53)	164 (51)
Extensive	81 (52)	77 (47)	158 (49)
			
*Performance status*			
0	10 (6)	5 (3)	15 (5)
1	88 (56)	52 (32)	140 (43)
2	39 (25)	77 (47)	116 (36)
3	15 (10)	29 (18)	44 (14)
4	4 (3)	1 (1)	5 (5)
Not known	1 (1)	1 (1)	2 (1)

**Table 2:** Demographic data and treatment modalities in patients with SCLC

	**No. of patients (%)**			
	**Age 70–74 years**	**Age 75–79 years**	**Age 80+ years**	**Total**
Patients	188 (58)	108 (34)	26 (8)	322

*Stage*				
Limited	85 (45)	60 (56)	19 (73)	164 (51)
Extensive	103 (55)	48 (44)	7 (27)	158 (49)

*PS*				
0	9 (5)	4 (4)	2 (8)	15 (5)
1	92 (49)	37 (34)	11 (42)	140 (43)
2	67 (36)	44 (41)	5 (19)	116 (36)
3	16 (9)	20 (19)	8 (31)	44 (14)
4	3 (3)	2 (2)	0	5 (2)
Not known	1 (1)	1 (1)	0	2 (1)

*Treatment*				
Cisplatin-containing regimes	32 (17)	13 (12)	3 (12)	48 (15)
Carboplatin combinations	92 (49)	49 (45)	11 (42)	152 (47)
Nonplatinum single agents	31 (17)	40 (40)	12 (46)	83 (26)
Other nonplatinum combinations	33 (6)	6 (6)	0	39 (12)

*Radiotherapy*				
Thoracic (within 3 months of chemo)	17 (9)	7 (6)	4 (15)	28 (8)
PCI	13 (7)	2 (2)	0	15 (5)

**Table 3:** Treatment modalities stratified by year of presentation in SCLC

	**No. of patient (%)**		
**Treatment**	**1982–1994**	**1995–2003**	**Total**
Cisplatin-containing regimes	16 (10)	32 (19)	48 (15)
Carboplatin combinations	59 (38)	93 (56)	152 (47)
Nonplatinum single agents	60 (38)	23 (14)	83 (26)
Other non-platinum combinations	22 (14)	17 (10)	39 (12)

*No. of cycles of chemotherapy*			
1	52 (33)	36 (22)	88 (27)
2	22 (14)	18 (11)	40 (12)
3	16 (10)	16 (10)	32 (10)
4	18 (11)	41 (25)	60 (19)
5	18 (11)	18 (11)	36 (11)
6+	31 (20)	36 (22)	67 (21)
Local radiotherapy	18 (11)	10 (6)	28 (9)
prophylactic cranial irradiation	3 (2)	12 (7)	15 (5)

**Table 4:** Survival differences among elderly SCLC patients between 1982–1994 and 1995–2003

**Year**	**1982–1994**	**1995–2003**	***P* values**
*Age 70–74 years*	Weeks	Weeks	
MS overall^a^	31	47	0.001
LD	33	60	0.03
ED	24	36	0.004
1-year survival	21%	44%	

*Age 75–80 years*			
MS overall^a^	25 weeks	28 weeks	0.06
LD	28	49	0.001
ED	27	27	0.7
1 year survival	6%	25%	

*Age* >*80 years*			
MS overall^a^	9 weeks	34 weeks	0.2
LD *n*=19	5	62	0.02
ED *n*=7	4	34	1.0
1-year survival	0%	30%	

MS=median survival.

a Results are adjusted for stage and PS.
